# Restoring the DREAM Complex Inhibits the Proliferation of High-Risk HPV Positive Human Cells

**DOI:** 10.3390/cancers13030489

**Published:** 2021-01-27

**Authors:** Claire D. James, Siddharth Saini, Fatmata Sesay, Kevin Ko, Jessica Felthousen-Rusbasan, Audra N. Iness, Tara Nulton, Brad Windle, Mikhail G. Dozmorov, Iain M. Morgan, Larisa Litovchick

**Affiliations:** 1 Philips Institute for Oral Health Research, School of Dentistry, Virginia Commonwealth University (VCU), Richmond, VA 23298, USA; cdjames@vcu.edu (C.D.J.); kok2@mymail.vcu.edu (K.K.); nultontj@protonmail.com (T.N.); bwindle@vcu.edu (B.W.); 2 Department of Internal Medicine, Division of Hematology, Oncology and Palliative Care, Virginia Commonwealth University (VCU), Richmond, VA 23298, USA; sainis2@mymail.vcu.edu (S.S.); sesayf@mymail.vcu.edu (F.S.); jfelthousen@mymail.vcu.edu (J.F.-R.); inessa2@mymail.vcu.edu (A.N.I.); 3 Massey Cancer Center, Virginia Commonwealth University (VCU), Richmond, VA 23298, USA; 4 Department of Biostatistics, Virginia Commonwealth University (VCU), Richmond, VA 23298, USA; mikhail.dozmorov@vcuhealth.org; 5 Department of Pathology, Virginia Commonwealth University (VCU), Richmond, VA 23298, USA

**Keywords:** human papillomavirus, oncogenic transformation, transcription, cell cycle, protein complex, head and neck cancer, cervical cancer, E7, DREAM

## Abstract

**Simple Summary:**

Human papillomaviruses are responsible for around 5% of all cancers, and to date there are no anti-viral therapeutics available for treating these cancers. In this report we demonstrate that in HPV positive cells the transcriptional repressor DREAM complex is disrupted by E7 proteins, with a resulting increase in expression of DREAM target genes. Expression of a mutant DREAM component, LIN52 S20C, competes with E7 and partially rescues DREAM complex formation. This restoration attenuates the growth of HPV positive cells, including HPV positive cervical cancer cell lines. We propose that restoration of the DREAM complex in HPV positive cancers is a novel therapeutic approach that could be adapted to aid in the treatment of these cancers.

**Abstract:**

High-risk (HR) human papillomaviruses are known causative agents in 5% of human cancers including cervical, ano-genital and head and neck carcinomas. In part, HR-HPV causes cancer by targeting host-cell tumor suppressors including retinoblastoma protein (pRb) and RB-like proteins p107 and p130. HR-HPV E7 uses a LxCxE motif to bind RB proteins, impairing their ability to control cell-cycle dependent transcription. E7 disrupts DREAM (Dimerization partner, RB-like, E2F and MuvB), a transcriptional repressor complex that can include p130 or p107, but not pRb, which regulates genes required for cell cycle progression. However, it is not known whether disruption of DREAM plays a significant role in HPV-driven tumorigenesis. In the DREAM complex, LIN52 is an adaptor that binds directly to p130 via an E7-like LxSxE motif. Replacement of the LxSxE sequence in LIN52 with LxCxE (LIN52-S20C) increases p130 binding and partially restores DREAM assembly in HPV-positive keratinocytes and human cervical cancer cells, inhibiting proliferation. Our findings demonstrate that disruption of the DREAM complex by E7 is an important process promoting cellular proliferation by HR-HPV. Restoration of the DREAM complex in HR-HPV positive cells may therefore have therapeutic benefits in HR-HPV positive cancers.

## 1. Introduction

Human papillomaviruses have been etiologically linked to 5% of all cancers worldwide [1]. In particular, the incidence of HPV-positive oropharyngeal cancers is steadily increasing, with high-risk (HR) type HPV16 responsible for 90% of these cases [2,3]. The HPV life cycle requires perturbation of the host cell cycle control machinery, mainly driven by the action of two viral oncogenes, E6 and E7 [4]. In HR-HPV, E6 promotes degradation of the “guardian of the genome” p53 [5]. E7 interacts with retinoblastoma (RB) family members including pRb, p107 and p130, also known as pocket proteins [6,7]. The RB family plays a key role in the G0/G1 arrest by repressing transcription of E2F-regulated cell cycle genes [8,9]. Previous studies revealed that pRb preferentially controls the function of the activator E2Fs 1, 2 and 3, whereas p130 and p107 serve as scaffolds to assemble multi-subunit DNA binding complexes that mediate the function of repressors E2F 4 and 5 (reviewed in [10,11,12,13]. HR-HPV E7 promotes degradation of pocket proteins and activation of E2F-mediated transcription, resulting in cell cycle entry [6]. Therefore, the consequences of E7 and E6 expression include an aberrant induction of cellular proliferation and genomic instability, which can lead to cancer. The molecular mechanisms involved in the early steps of HPV cancer pathogenesis are not fully understood.

The interaction of HR-HPV E7 proteins with RB family members requires an LxCxE motif in the viral protein [6]. Other viral oncoproteins such as SV40 Large T and adenovirus E1 also use an LxCxE motif to bind to a conserved cleft in the RB protein family [14,15]. Recent studies revealed that the LxCxE-binding domain of the RB-like proteins p107 and p130 mediates the assembly of a multi-subunit DNA binding complex called DREAM [11]. DREAM is an evolutionarily conserved transcriptional repressor composed of DP, Rb-like, E2F and MuvB core, which includes five proteins related to *C. elegans* multi-vulva class B (MuvB) genes LIN9, LIN37, LIN52, LIN53/RBBP4 and LIN54 (reviewed in [10]) [11,16,17]. The MuvB core plays a dual role in regulation of cell cycle gene expression in mammalian cells. In G0/G1, it binds p130 to form DREAM and repress transcription of cell-cycle regulated genes. Phosphorylation of LIN52 at S28 by dual specificity tyrosine (Y)-regulated kinase A1 (DYRK1A) is required for p130 binding and DREAM complex assembly [18,19]. In S-phase, MuvB core leaves DREAM and interacts with the B-Myb transcription factor to form the Myb-MuvB (MMB) complex required for G2/M gene expression [11,12,13]. Overexpression of B-Myb (encoded by *MYBL2*, a DREAM target gene) disrupts the DREAM complex in vitro and correlates with poor clinical outcomes in breast and other cancers [20,21].

Only p130 and p107, but not pRb, interact with the MuvB core to form DREAM [11]. This specificity is due to the LIN52 subunit of MuvB that serves as an adaptor for RB-like proteins but does not bind pRb. Crystallography studies revealed that the N-terminus of LIN52 binds to the LxCxE-binding cleft of p107 and p130, while its C-terminus interacts with B-Myb [18,22]. LIN52 is a small protein of 116 amino acids, and this could constrain simultaneous binding of p130 and B-Myb. In addition to phosphorylated S28, p107 and p130-binding by LIN52 requires a LxSxE sequence, similar to the LxCxE motif in HR-HPV E7 [18,19]. Comparison of the crystal structure of the p107-E7 and p107-LIN52 complexes revealed subtle but important differences, resulting in a more stable interaction with E7 than LIN52. Due to its higher binding affinity, E7 displaces LIN52 from the p130 “pocket”, causing DREAM disassembly and MMB complex formation (Figure S1). Previously, we found that a LIN52 mutant, LIN52-S20C, that reconstitutes the LxCxE motif, can compete with E1A and SV40 Large T for binding to p130 [18]. 

Transgenic mice expressing HPV16 E7 under the keratin-specific promoter develop carcinoma tumors in oral carcinogen studies [23,24]. pRb knock out mice treated in the same way had epithelial hyperplasia and improper differentiation but not carcinoma formation [25,26]. Importantly, the combinations of pRb with p107 or p130 knock out resulted in increased oral carcinogenesis, demonstrating the important contribution of p107/p130 to cancer development [27,28]. These observations motivated us to investigate the role of DREAM (a complex that includes p107 or p130, but not pRb) in HR-HPV positive epithelial cells. We found that E7 disrupts DREAM and promotes MMB complex formation, whereas the expression of LIN52-S20C (which competes with E7 for p107/p130 binding) partially rescues DREAM assembly in human cells harboring the HR-HPV genome, resulting in attenuation of cellular growth. These findings establish the DREAM complex as an important target of HR-HPV-mediated oncogenic transformation independent of the pRb and p53 tumor suppressors.

## 2. Results

### 2.1. MMB and DREAM Target Genes Are Upregulated by HPV16

**Figure 1:**
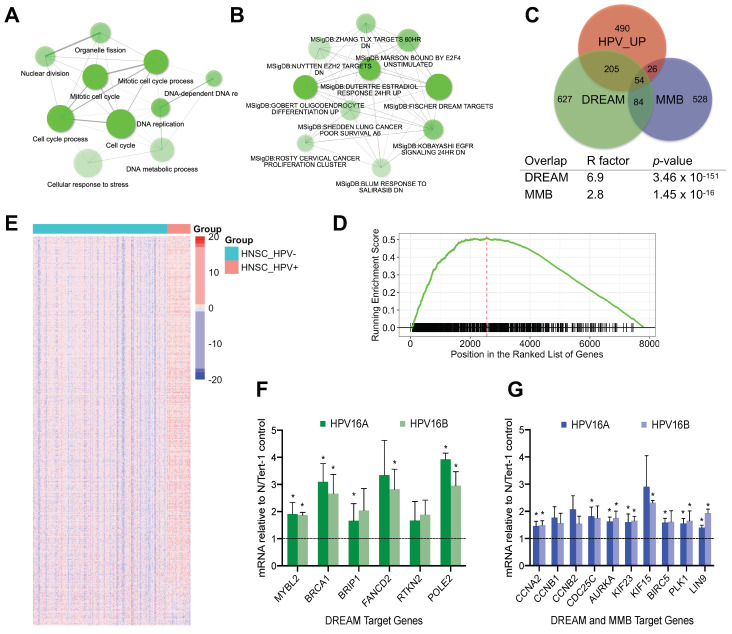
Oncogenic HPV disrupts DREAM-regulated transcription. (**A**). Top ten enriched GO: BP terms in the N/Tert-1+HPV16 vs. control cells comparison. Nodes that share 20% or more genes are connected; thicker edges and bigger nodes correspond to more genes, and brightness reflects the enrichment rank. (**B**). Top ten enriched curated gene sets (MSigDB) among the genes upregulated by HPV16 in N/Tert-1 cells. (**C**). Proportional Venn diagram of the HPV16-upregulated genes, DREAM and MMB targets. Representation factor (R) is the number of the resultant genes in the overlap divided by expected. (**D**). GSEA shows enrichment of the HPV+ *vs*. negative differentially expressed genes, TCGA HNSC tumor samples in the DREAM target gene set. (**E**). Heatmap shows expression of the DREAM target genes (rows) in TCGA HNSC samples, separated by HPV16 status. (**F**,**G**). RT-qPCR analysis of select DREAM and MMB target genes in two N/Tert-1+HPV16 cell lines compared to control (set as 1, dashed line). Graphs here and below show mean ± SD of 3 repeats; * *p* < 0.05 (*t*-test) indicates values significantly different from the control N-Tert1 cells.

Previously, we established a HPV16 life cycle model in the immortalized human foreskin keratinocyte cell line N/Tert-1 and used RNA-seq to identify transcriptome changes induced by HPV16 in these cells [29,30]. Gene ontology analysis of differentially expressed genes (DEGs) revealed that HPV16-impacted pathways are involved in cell cycle progression (Figure 1A). Furthermore, gene set enrichment analysis of HPV16-upregulated genes identified direct targets of the DREAM complex among the top ten gene sets (Figure 1B and Table S1) [10,11,29,31]. DREAM binds to and represses cell-cycle dependent genes expressed in both the G1/S and G2/M phases, while MMB-regulated genes include the genes expressed in G2/M [10,19,31]. We found that both direct targets of the DREAM (bound by p130 or E2F4, and MuvB core subunits) and MMB (bound by B-Myb and MuvB) according to the TargetGeneReg database [11,12,13] were over-represented among HPV16-upegulated genes (Figure 1C).

Next, we investigated whether the effects of HPV on DREAM target gene expression could be observed in human tumor samples. Gene set enrichment analysis (GSEA) of HPV-positive and negative head and neck cancer (HNC) TCGA samples revealed significant enrichment of the DREAM target genes in the differentially expressed gene sets (Figure 1D, GSEA *p*-value = 1.00 × 10^−10^), as well as their upregulation in HPV+ tumors (Figure 1E). This effect was confirmed using RT-qPCR analysis of selected DREAM and MMB target genes in two clonal lines of N/Tert-1+HPV16 cells when compared with N/Tert-1 (Figure 1F,G).

### 2.2. HPV16 E7 Associates with p130 and Alters the DREAM-MMB Balance in N/Tert-1 Cells

**Figure 2:**
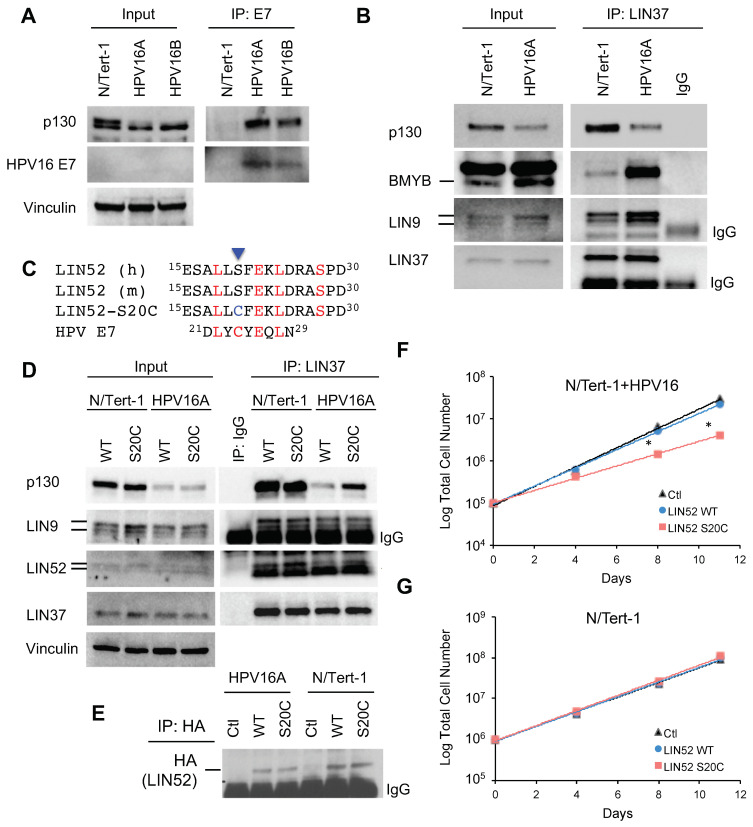
HPV16 E7 disrupts DREAM by displacing LIN52. (**A**). IP/WB assay shows interaction between p130 and E7 in N/Tert-1+HPV16 cell lines. (**B**). IP/WB analysis of DREAM and MMB complexes in N/Tert-1 cell lines. Specific bands are indicated by lines. (**C**). Protein sequence of LIN52 aligned with E7 LXCXE motif. Arrowhead shows residue mutated in LIN52-S20C; pS is the DYRK1A phosphorylation site. (**D**). IP/WB assay analysis of DREAM complex in the indicated cell lines. (**E**). Expression of LIN52 alleles detected using IP and WB with anti-HA antibodies. (**F**,**G**). Cell proliferation assay with indicated cell lines. * *p* < 0.05 (*t*-test) indicates significant difference with the control cell line.

We next assessed the status of the DREAM and MMB complexes in N/Tert-1+HPV16 cells. As expected, we found that p130 interacts with E7 in these cells, and that p130 levels are reduced in HPV16+ cells compared to control (Figure 2A). The interaction of LIN37 with p130 was decreased, and its binding to B-Myb was increased by HPV16, demonstrating a switch from DREAM (p130) to MMB (B-Myb) formation (Figure 2B). We also observed increased levels of B-Myb and LIN9 proteins in the presence of HPV16, consistent with increased expression of the *MYBL2* and *LIN9* genes (Figure 1F). Overall, these findings demonstrate disruption of the DREAM complex in N/Tert-1+HPV16 cells.

### 2.3. Expression of LIN52-S20C Partially Overcomes the Effect of HPV16 in N/Tert-1+HPV16 Cells

LIN52 binds to p130 directly through the sequence LxSxE and the adjacent phosphorylated S28 residue, and mutation of serine 20 in LIN52 to a cysteine (LIN52-S20C) increases its affinity to p130 (Figure 2C) (16). To test whether LIN52-S20C can selectively rescue DREAM assembly in the presence of HPV16, we generated stable N/Tert-1 and N/Tert-1+HPV16 cell lines expressing Flag-HA-tagged wild type LIN52 (WT) or LIN52-S20C mutation (S20C), using retroviral delivery. We assessed the status of DREAM and MMB complexes in these cell lines using LIN37 pull-down and detection of p130 (Figure 2D). Although LIN52-S20C had no effect on DREAM complex formation in the control N/Tert-1 cells when compared with WT-LIN52, this mutant increased DREAM assembly in the N/Tert-1+HPV16 cells as evident by increased LIN37-p130 interaction in the cells expressing the LIN52-S20C compared to WT cells. The exogenous LIN52 expression in these cell lines was below detection levels using direct anti-HA immunoblotting but could be confirmed using anti-HA IP and western blot (Figure 2E). The levels of p130 remained lower in N-Tert-1+HPV16 cells than in control with LIN52-S20C expression, likely because of the incomplete disruption of E7-p130 interaction. Consistent with its ability to rescue DREAM assembly, LIN52-S20C significantly decreased proliferation of the N/Tert-1+HPV16 cells, whereas WT-LIN52 had no effect (Figure 2F). Importantly, proliferation of the control N/Tert-1 cells was not influenced by expression of these LIN52 proteins (Figure 2G).

### 2.4. Expression of S20C-LIN52 Partially Rescues DREAM in HR-HPV Cancer Cell Lines

**Figure 3:**
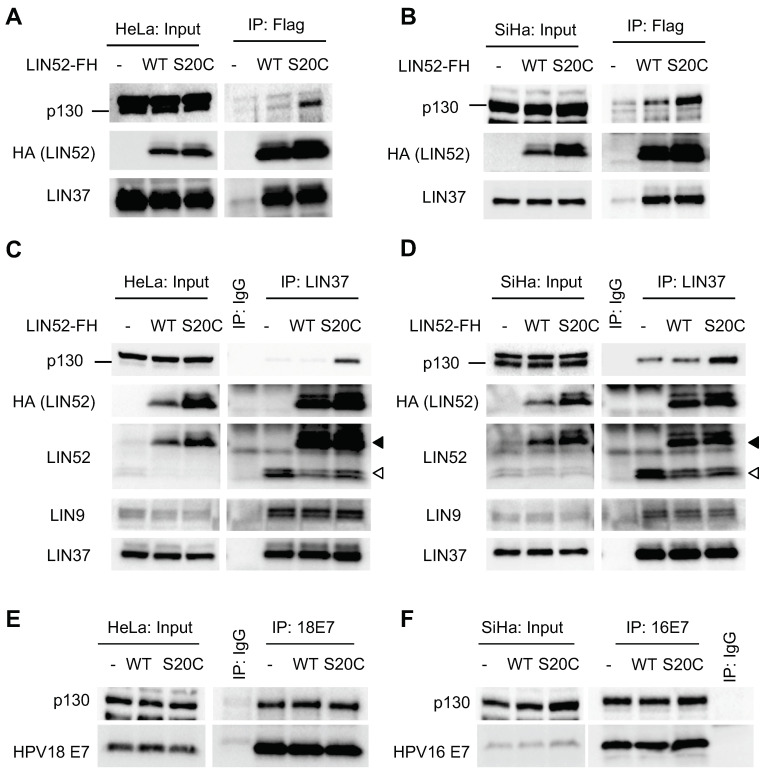
LIN52-S20C rescues DREAM in HR-HPV cancer cell lines. (**A**,**C**). IP/WB assays show increased binding between p130 and MuvB components in LIN52-S20C-expressing HeLa cells compared to controls. Black and white arrowheads mark the recombinant and endogenous LIN52 bands, respectively. (**B**,**D**). The same assays with SiHa cells. (**E**,**F**). IP/WB assays show similar binding between p130 and E7 in HeLa (**E**) and SiHa (**F**) cell lines.

To determine whether LIN52-S20C can restore DREAM in HPV-positive cancer cells, we stably expressed WT-LIN52 or LIN52-S20C in human cervical cancer cell lines HeLa (HPV18) and SiHa (HPV16). Again, the binding of p130 to LIN52-S20C in these cells was increased compared to WT-LIN52 (Figure 3A,B). This effect was specific because both forms of LIN52 immunoprecipitated equal amount of MuvB core protein LIN37. Furthermore, expression of LIN52-S20C increased the DREAM assembly in HeLa and SiHa cells as LIN37 interacted more with p130 in the presence of LIN52 S20C, whereas the wild-type LIN52 had no effect on the p130-LIN37 interaction (Figure 3C,D). Similar amounts of LIN9 and LIN52 (both endogenous and overexpressed) were bound to LIN37, confirming the specificity of this effect. Similar to N/Tert-1+HPV16 cells, LIN52-S20C had no significant effect on p130 levels in these cells. Furthermore, the amount of p130 co-precipitated by E7 from HeLa and SiHa cells was unchanged by LIN52-S20C or the wild type LIN52 expression, indicating incomplete rescue of DREAM by LIN52-S20C (Figure 3E,F). 

### 2.5. Expression of LIN52-S20C Inhibits Growth of HR-HPV Cancer Cell Lines 

**Figure 4:**
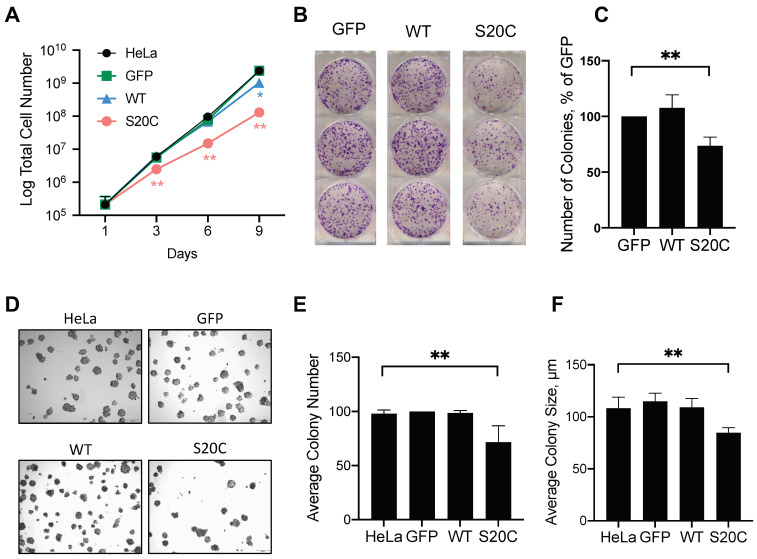
LIN52-S20C attenuates growth of HeLa cells. (**A**). Cell proliferation assay with indicated cell lines. * *p* < 0.05; ** *p* < 0.01 (2-tailed paired *t*-test for each time point) indicate significant differences with control (HeLa) cell line. (**B**–**F**). Representative images and quantification of the clonogenic assays (**B**,**C**) and anchorage-independent growth assays (**D**–**F**) with indicated HeLa cell lines. ** *p* < 0.01 (ANOVA with Dunnett’s multiple comparisons test) indicates significant difference between LIN52-S20C and control (HeLa) cell lines. There was no significant difference between the parental HeLa cells and either GFP-expressing or WT LIN52 HeLa cell lines (*p* > 0.05).

To investigate the functional consequences of the partial restoration of the DREAM by LIN52-S20C, we measured HeLa cell proliferation and colony formation using both adherent and anchorage-independent culture conditions. Expression of LIN52-S20C significantly slowed the growth of HeLa cells, compared to control and the WT-LIN52 (Figure 4A). In addition, the S20C expressing cells formed significantly fewer colonies after single-cell plating, both on plastic (Figure 4B,C) and in methylcellulose (Figure 4D–F). Furthermore, the size of the S20C colonies formed in methylcellulose was significantly smaller than in WT or control cells (Figure 4F). Similar results were also seen in SiHa cells (Figure S2). Consistent with restoring DREAM-mediated repression, several DREAM target genes including *CCNA2*, *CCNB1* and *PLK1* were expressed at reduced levels in HeLa cells expressing LIN52-S20C compared to control cells while the expression of other genes was not repressed (Figure S3). 

It is possible that expression of LIN52-S20C could indirectly re-activate the function of other tumor suppressors targeted by HPV in cancer cells. However, we found no evidence that the functional status of pRb and p53 tumor suppressors was different in the WT and LIN52-S20C HeLa cell lines (Figure S4). Therefore, the data presented here support the model that partial restoration of DREAM by LIN52-S20C could inhibit proliferation of HPV+ cancer cells. 

In conclusion, we found that oncogenic E7 interacts with p130 when expressed at physiologically relevant levels in HPV-positive cells. Furthermore, our data demonstrates that E7 competes with the MuvB core component LIN52 to bind p130 via an LxCxE-like binding motif, resulting in disassembly of the DREAM complex, deregulation of cell cycle gene expression and increased cell proliferation. The E7-competitive mutant LIN52-S20C is able to partially rescue DREAM complex assembly and suppress proliferation of the cells driven by HPV16 and HPV18, supporting the role of DREAM as an important HPV oncogenic target.

## 3. Discussion

The cancer driver effect of HR-HPV provides a unique opportunity for anti-viral targeted therapy. To date, no such anti-viral therapeutics exist, and to develop them we need a better understanding of viral-host interactions and the mechanisms that contribute to oncogenesis. E7 is a major oncogene of all HR-HPV, its actions disrupt host gene transcription and induce DNA damage in the infected cell [6,7,32,33,34,35]. A major determinant of E7 disruption of host gene transcription is mediated via interaction with the pocket proteins pRb, p107 and p130. These pocket proteins interact with E2F transcription factors and repress their activation potential, whereas E7 alleviates this repression resulting in E2F target gene activation. Recent studies revealed distinct molecular mechanisms by which the pocket proteins coordinate the expression of cell cycle regulated genes (reviewed in [19]). However, the precise contribution of the pocket proteins to E7 mediated cell proliferation and oncogenesis remains to be fully elucidated. 

Transgenic mouse models using K14 (Keratin 14) driven expression of E7 provided several key insights into E7 oncogenesis. The K14-E7 mice displayed epidermal hyperplasia in multiple sites, including skin, mouth palate, esophagus, forestomach and ectocervix [36]. Development of these phenotypes required intact CR2, a region of E7 that binds to pocket proteins [37]. Furthermore, estradiol treatment of the K14-E7 mice resulted in cervical cancers [38,39], while addition of the carcinogen 4NQO into their diet caused accelerated oral carcinogenesis [39]. Genetic deletion of pRb in keratinocytes only partially recapitulated the E7 phenotype with epithelial hyperplasia without tumor formation. Importantly, expression of E7 in either pRb knockout animals, or in the presence of a pRb mutant resistant to E7 targeting, still resulted in tumorigenesis [26,40]. Finally, the combinations of pRb with p107 or p130 knock out resulted in increased oral carcinogenesis compared with individual deletions [27,28]. These results demonstrate that inactivation of all pocket proteins by the oncogenic CR2 domain of E7 contributes to cancer progression.

Given our previous work demonstrating the critical role of the DREAM complex (which incorporates p107 and p130, but not pRb) in regulating cell-cycle dependent gene expression, we investigated the mechanism of DREAM complex disruption in HR-HPV positive cells. Here we demonstrated that introduction of HR-HPV genome disrupts the DREAM complex in human keratinocytes by dissociation of MuvB core from p130, resulting in elevated expression of DREAM and MMB target genes. This finding is in agreement with previous reports of the DREAM disruption in human and mouse cell lines with overexpression of E7 protein [41,42]. Using a LIN52-S20C mutant that competes with the LxCxE motif of viral oncoproteins [16], we were able to partially rescue the DREAM complex assembly in the presence of HR-HPV. Increased assembly of DREAM resulted in decreased proliferation of the HR-HPV+ keratinocytes and cervical cancer cell lines, demonstrating the role of DREAM complex as an important target of HR-HPV to promote carcinogenesis. Furthermore, LIN52-S20C did not affect the growth on non-HPV cell lines, in support of the specific effect of disruption of DREAM on proliferation of HPV-positive cells. Expression of LIN52-S20C in HR-HPV cell lines did not result in complete growth arrest, as in case of E7 depletion [41,42], most likely due to an incomplete rescue of the DREAM complexes in the cell. This reason could also explain observed incomplete repression of DREAM-target genes in the S20C-LIN52 HeLa cells. Since this analysis was limited to only a small group of genes, further transcriptome studies will be required to characterize the effect of DREAM restoration on cell-cycle regulated gene expression. Notably, the attenuation of cell proliferation occurs even in the presence of E7-mediated inactivation of pRb that is not affected by the expression of the LIN52 S20C mutant. This finding could provide a rationale for future studies aimed to stabilize the LIN52-p130 interaction for control of HPV+ cancer cell proliferation. 

Overall, the results present evidence that disruption of the DREAM complex plays an important role in HR-HPV induced cell proliferation. Future studies will focus on modeling our results in transgenic mice, and also on identifying the LIN52 modifications for more efficient disruption of the interaction between E7 and p107/p130, resulting in complete restoration of the DREAM complex. The ability to control proliferation of the HR-HPV-positive cells without affecting “normal” cells could lead to development of virus-specific cancer treatments.

## 4. Materials and Methods

### 4.1. Cell Culture

Clonal cell lines containing the HPV16 genome were generated from telomerase-immortalized human keratinocytes (N/Tert-1) and grown as previously described [29]. HeLa, SiHa and U-2 OS cells (ATCC) were grown as recommended by vendor. Cell lines expressing Flag-HA-tagged GFP (control), wild-type LIN52 or LIN52-S20C were generated by retroviral delivery of vectors followed by selection with puromycin (1 μg/mL, Sigma-Aldrich, St. Louis, MO, USA) as previously described [17]. 

All cell-based assays included 3 biological repeats. Quantification data were analyzed and plotted using Prism software version 8 (GraphPad Software, San Diego, CA, USA). Statistical significance for comparisons with control was determined using Prism 8, either with Student’s 2-tailed *t*-test, or with ANOVA and Dunnett’s post hoc correction for multiple comparisons, as indicated in figure legends. To measure proliferation, 3 × 10^5^ cells were seeded in triplicate onto 10 cm dishes, grown for 3 days, then detached, and counted using trypan blue staining. Total number of cells was recorded, and 3 × 10^5^ cells per dish were re-plated each time on days 3 and 6. For clonogenic assays, 10^3^ cells were seeded in triplicate wells of a 6-well plate, and colonies were grown for 10 days, stained with Crystal Violet dye and quantified using ImageJ software (National Institutes of Health, Bethesda, MD, USA) [43]. The anchorage-independent growth assay was adapted from [44]. Briefly, 5 × 10^3^ cells were seeded in triplicate wells in a 24-well ultra-low attachment plate (Corning Inc., Corning, NY, USA) in growth medium containing 1% methylcellulose, incubated for 14 days and imaged. The colony diameter and number of colonies > 30 μm per 100 visible cells were measured using ImageJ.

### 4.2. RNA-seq Data Analysis

TCGA RNAseq data (PanCancer Atlas) for HPV+ and HPV- HNC squamous cell carcinoma samples [45] were analyzed using cBio portal [46,47]. DEGs from TCGA data analysis as well as our gene expression data from N/Tert-1 cell lines [29,30] were analyzed using ShinyGo V6.01 tool [48]. Statistical analysis of gene overlap was done using tools at Nemates.org. The clusterProfiler v.3.16.0 R package was used to perform GSEA. The log2(TPM + 1) gene expression values were used to plot the heatmap using the heatmap v.1.0.12 R package.

### 4.3. mRNA and Protein Analysis

SYBR Green RT-qPCR gene expression analysis was done as described using primers from Table S2 [29,30]. Immunoblotting and immunoprecipitations were performed as described [12,17]. Briefly, cells were rinsed and scraped with cold PBS h containing protease and phosphatase inhibitor cocktails (EMD Chemicals, San Diego, CA, USA). Cells were extracted with EBC buffer (Boston BioProducts, Ashland, MA, USA) supplemented with protease and phosphatase inhibitors and 1.4 mM β-mercaptoethanol. Clarified extracts were incubated with 1 μg of capturing antibody and protein A Sepharose CL-4B beads (GE Healthcare, Chicago, IL, USA) overnight at 4 °C. The input extracts and immunoprecipitated samples were resolved using 4–20% gradient Criterion SDS-PAGE gels (BioRad, Hercules, CA, USA), and transferred to nitrocellulose membranes (GE Healthcare). Proteins were detected with commercial primary and secondary antibodies listed in Table S3, and with previously described antibodies against MuvB complex components LIN52 (12 kDa), LIN37 (40 kDa) and LIN9 (65 kDa) [12,17]. 

## 5. Conclusions

In conclusion, we found that oncogenic E7 interacts with p130 when expressed at physiologically relevant levels in HPV-positive cells. Furthermore, our data demonstrates the mechanisms by which E7 competes with the MuvB core component LIN52 to bind p130 via an LxCxE-like binding motif, resulting in disassembly of the DREAM complex, deregulation of cell cycle gene expression and increased cell proliferation. The E7-competitive mutant LIN52-S20C is able to partially rescue DREAM complex assembly and suppress proliferation of the cells driven by HPV16 and HPV18, supporting the role of DREAM as an important HPV oncogenic target. Reversal of E7 disruption of DREAM is a novel therapeutic target for combating HPV cancers.

## Data Availability

The data presented in this study are available in this article (and supplementary material).

## Supplementary Materials

The following are available online at https://www.mdpi.com/2072-6694/13/3/489/s1. Table S1: Curated MSigDB enriched Genes r, Table S2: Primer sequences, Table S3: Commercial antibodies, Figure S1: Schematic presentation of the mechanism by which high-risk HPV E7 proteins disrupt the DREAM complex, Figure S2: LIN52-S20C attenuates growth of SiHa cells, Figure S3: Effect of LIN52 and LIN52-S20C on cell cycle gene expression, Figure S4: LIN52-S20C does not restore the function of pRb and p53.
