# Supplementary material for: Restoring the DREAM Complex Inhibits the Proliferation of High-Risk HPV Positive Human Cells
Source: Cancers (Basel). 2021 Jan 27;13(3):489. doi: 10.3390/cancers13030489 (PMC7866234; doi:10.3390/cancers13030489)

cancers MDPI

Restoring the DREAM Complex Inhibits the Proliferation of High-Risk HPV Positive Human Cells

Claire D. James, Siddharth Saini, Fatmata Sesay, Kevin Ko, Jessica Felthousen-Rusbasan, Audra N. Iness, Tara Nulton, Brad Windle, Mikhail G. Dozmorov, Iain M. Morgan and Larisa Litovchick

Table S2. Primer sequences.

Target	Forward (5′-3′)	Reverse (5′-3′)	Size (bp)
CCNA2	TGCTGGAGCTGCCTTTCATTTAGCA	ACTGTTGTGCATGCTGTGGTGC	158
PLK1	TCCAAGCCCTCGGAGCGTGG	ACCCAAGGCCGTACTTGTCCG	115
MYBL2	CATTGTGGATGAGGATGTGAAGC	TGGTTGAGCAAGCTGTTGTCTTC	132
BIRC5	AGGACCACCGCATCTCTACAT	AAGTCTGGCTCGTTCTCAGTG	118
AURKA	GGAATATGCACCACTTGGAACA	TAAGACAGGGCATTTGCCAAT	108
KIF23	TGTGGCTAATCCCTTGGTCAA	AGAACCAGTCATTGTGTGAGTTT	103
CDC25C	ATGACAATGGAAACTTGGTGGAC	GGAGCGATATAGGCCACTTCTG	185
CCNB2	CCGACGGTGTCCAGTGATTT	TGTTGTTTTGGTGGGTTGAACT	180
LIN9	GGAACGAAAGTTACAGCACGA	CAAGCCCTGTCCTATCAAAAGT	118
CCNB1	AAGAGCTTTAAACTTTGGTCTGGG	CTTTGTAAGTCCTTGATTTACCATG	319
BRCA1	ACCTTGGAACTGTGAGAACTCT	TCTTGATCTCCCACACTGCAATA	136
BRIP1	CTTACCCGTCACAGCTTGCTA	CACTAAGAGATTGTTGCCATGCT	144
FANCD2	AAAACGGGAGAGAGTCAGAATCA	ACGCTCACAAGACAAAAGGCA	180
KIF15	CTGAAGCCTATCAGGTGTTGTC	AGGGAGGTCCGTATATTCACAAT	154
RTKN2	ATGCTCGACTAATGGCCTATACA	CGTCGTGATCGTTCTTTATTGCT	196
POLE2	ATTTACTCCTCCGGTGATAGGTT	GCATCTCCGATTTTGGTTGTACT	105
GAPDH	GGAGCGAGATCCCTCCAAAAT	GGCTGTTGTCATACTTCTCATGG	197

Table S3. Commercial antibodies.

Protein	MW, kDa	Vendor (Catalog #)	Use
Vinculin	120	Sigma (V9131)	WB loading control (1:10000)
GAPDH	37	Millipore (MAB374)	WB loading control (1:10000)
p130	150	BD Biosciences (610262)<br>Santa Cruz (SC-317)	WB Fig. 2A&B, 3E&F (1:3000)<br>WB Fig. 2B&D, 3C&D (1:10000)
B-Myb	80	Millipore (MABE886)	WB Fig. 2B (1:1000)
pRb	110	Cell Signaling (9309)	WB Fig. S3 (1:1000)
E2F1	70	Cell Signaling (3742)	IP/WB Fig. S3 (1:1000)
p53	53	Santa Cruz (DO1, sc-126)	WB Fig. S3 (1:1000)
p21	21	BD Pharmingen (556331)	WB Fig. S3 (1:1000)
HPV16 E7	20	Santa Cruz (SC-65711)	IP/WB Fig. 2A, 3E (1:1000)
HPV18 E7	20	Santa Cruz (SC-365035)	IP/WB Fig. 3F (1:1000)
HA		Cell Signaling (3724)	IP/WB Fig. 2E (1:1000); WB Fig. 3ABCD
Flag		Sigma (F1804)	IP and WB Fig. 3A&B (1:3000)
Anti-Rabbit IgG (H+L) HRP		Jackson<br>(711-005-152)	Secondary for WB with rabbit antibodies (1:5000)
Anti-Rabbit IgG (L) HRP		Jackson<br>(211-032-171)	WB detection of IP with rabbit antibodies (1:1000)
Anti-Mouse HRP		Jackson<br>(715-035-150)	Secondary for WB with mouse antibodies(1:5000)

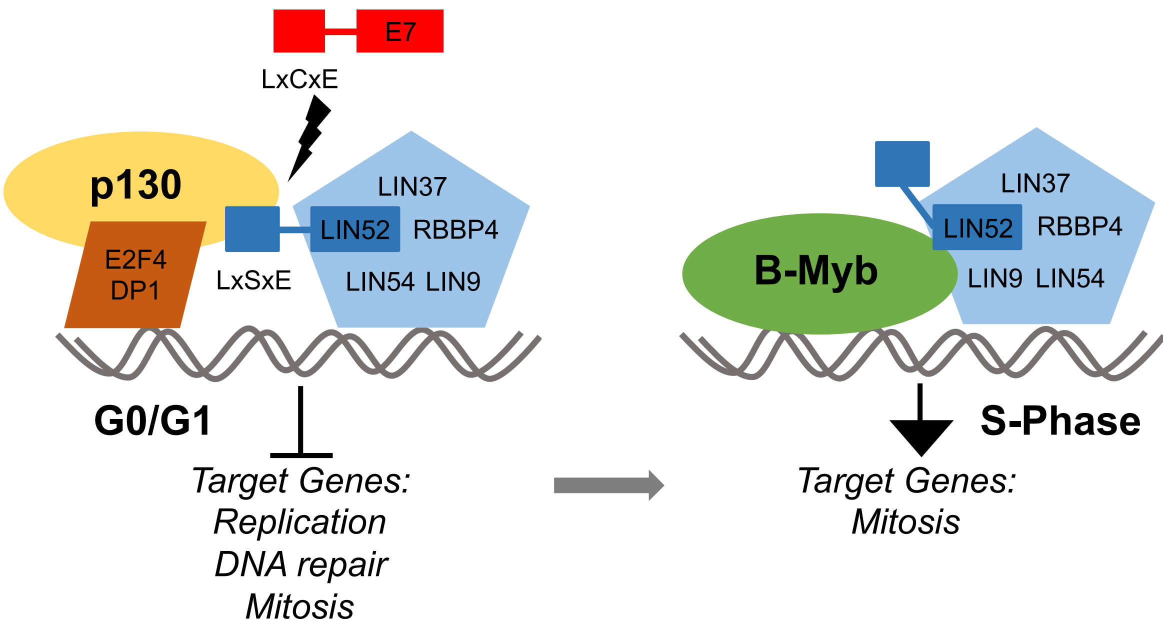

Figure S1. Schematic presentation of the mechanism by which high-risk HPV E7 proteins disrupt the DREAM complex.

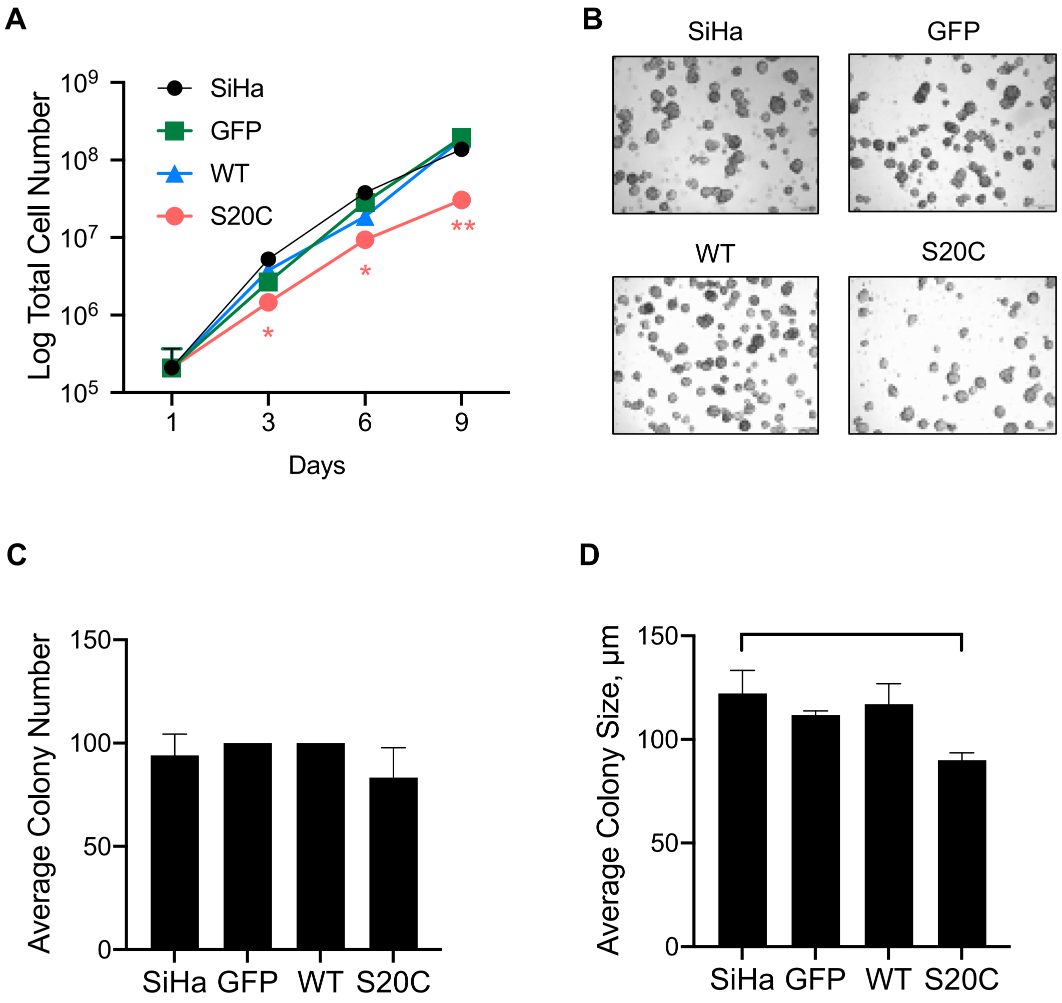

Figure S2. LIN52-S20C attenuates growth of SiHa cells. A. Cell proliferation assay with indicated SiHa cell lines. **B-D.** Representative images and quantification of the anchorage-independent growth assays with indicated SiHa cell lines. ** - p<0.01 (ANOVA with Dunnett's multiple comparison tests, each group was compared to control SiHa cell line).

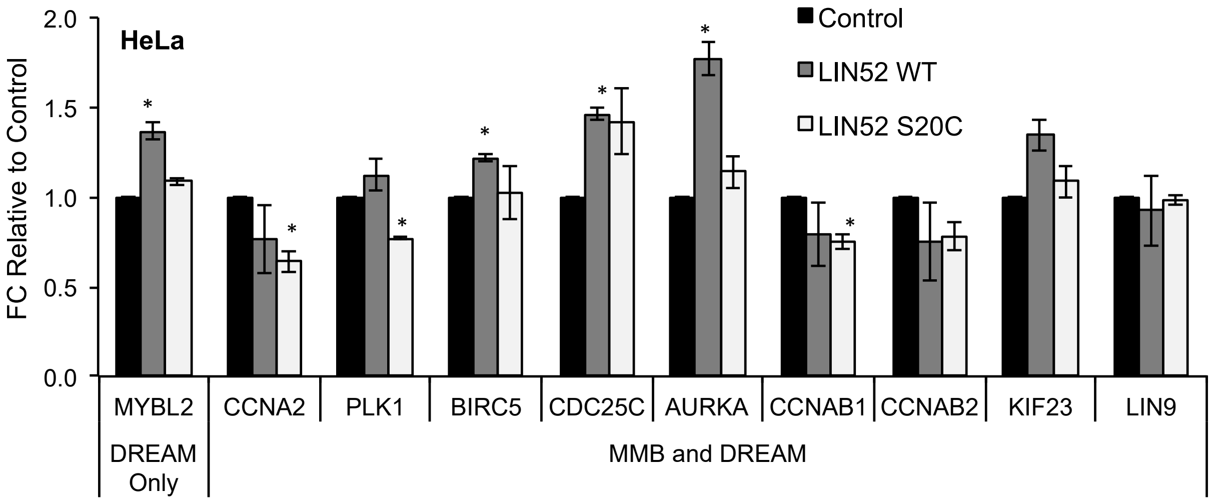

Figure S3. Effect of LIN52 and LIN52-S20C on cell cycle gene expression. HeLa stable cell lines expressing GFP (Control), wild type LIN52 (WT) or LIN52-S20C mutants were used for RT-qPCR analysis of the indicated genes.

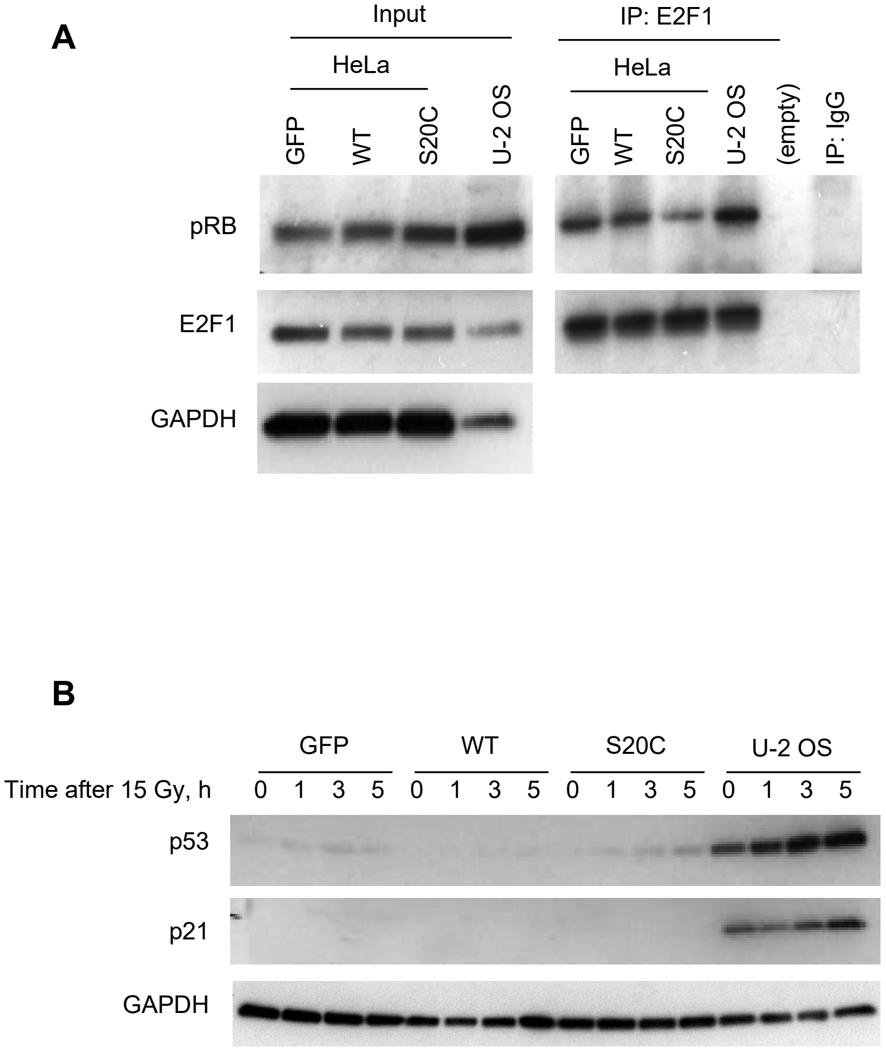

Figure S4. LIN52-C20C does not restore the function of pRb and p53. A. IP-WB assay with indicated HeLa cell lines, and U-2 OS cell line with intact pRb as a control. **B.** Indicated HeLa cell lines along with U-2 OS control cells were treated with -irradiation (15 Gy) and analyzed for induction of indicated proteins using WB. GAPDH serves as loading control.

Fig. 2A (uncropped)

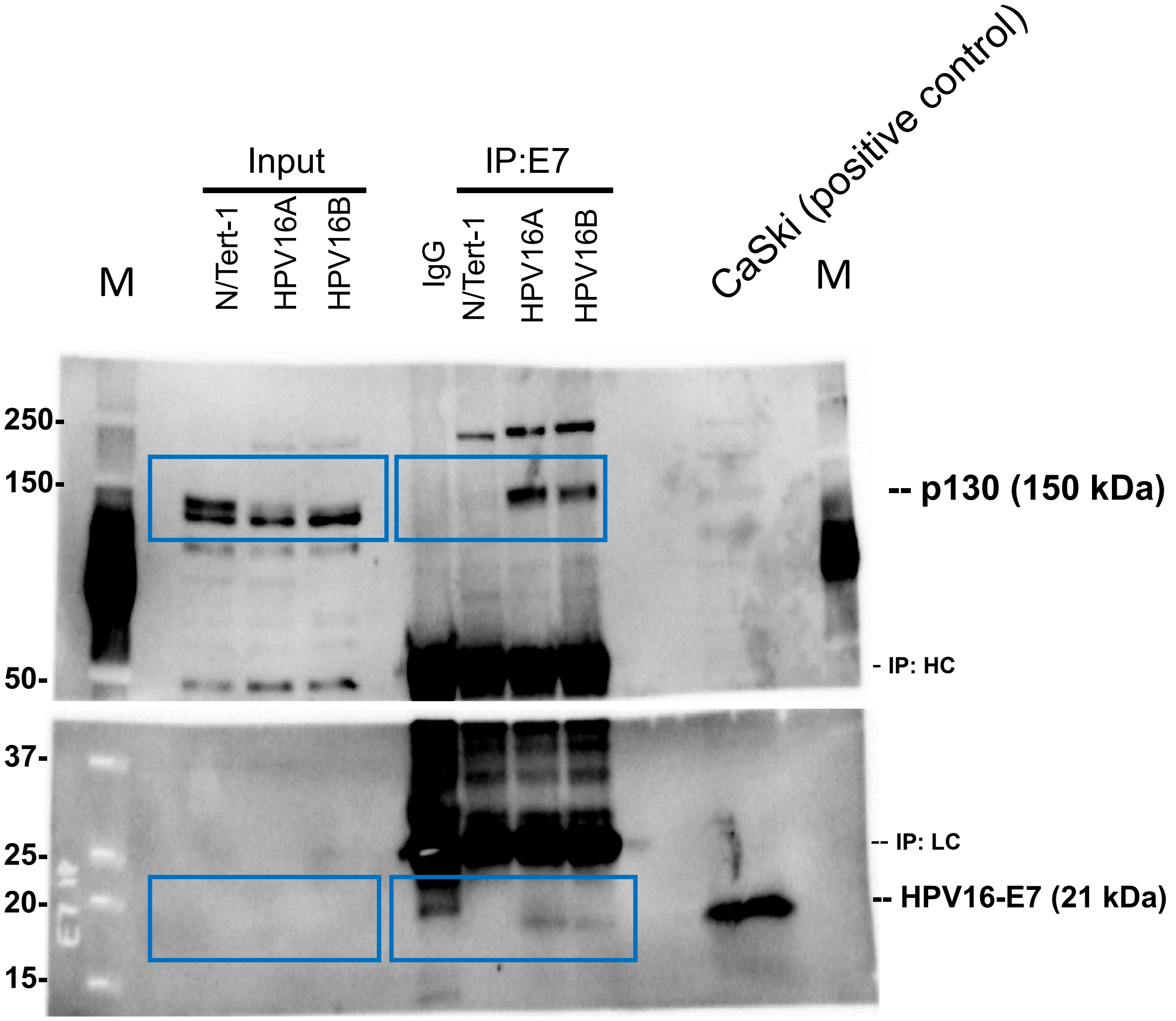

Fig. 2B (uncropped)

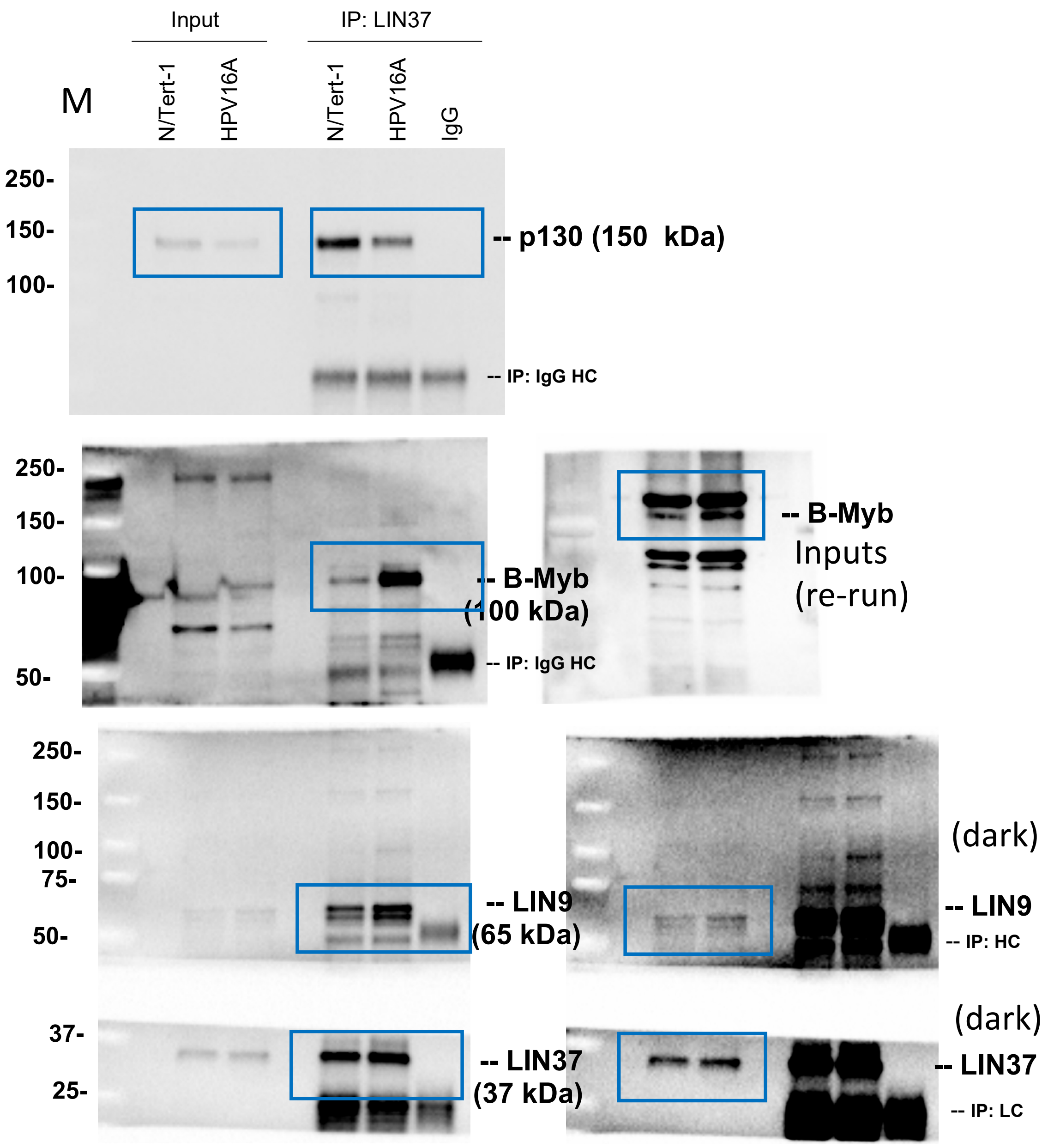

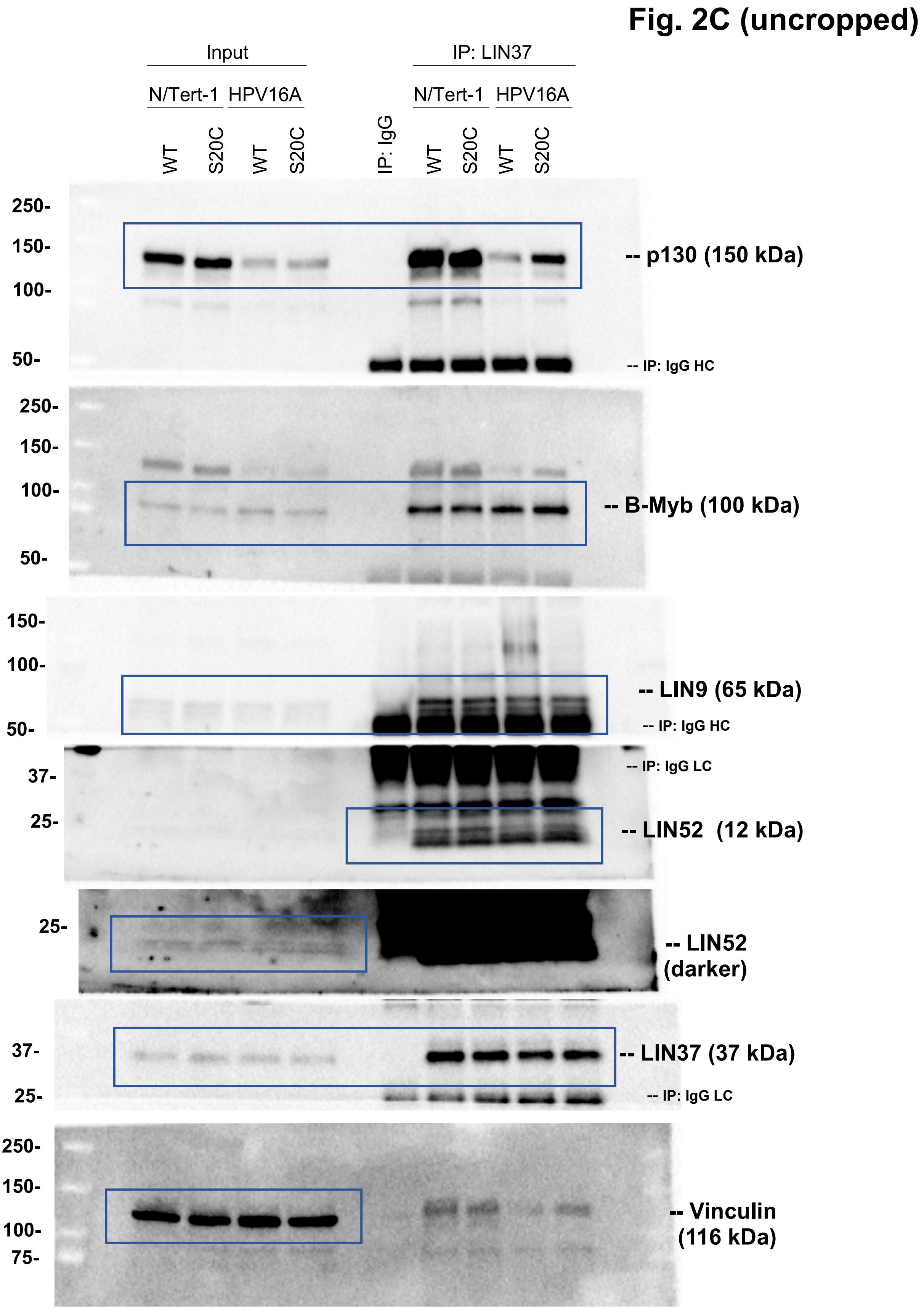
Fig. 2C (uncropped)
Input
IP: LIN37
N/Tert-1
HPV16A
N/Tert-1
HPV16A
WT
S20C
WT
S20C
IP: IgG
WT
S20C
WT
S20C
250-
150-
100-
50-
-- p130 (150 kDa)
-- IP: IgG HC
250-
150-
100-
50-
-- B-Myb (100 kDa)
150-
100-
50-
-- LIN9 (65 kDa)
-- IP: IgG HC
-- IP: IgG LC
37-
25-
-- LIN52 (12 kDa)
25-
-- LIN52 (darker)
37-
25-
-- LIN37 (37 kDa)
-- IP: IgG LC
250-
150-
100-
75-
-- Vinculin (116 kDa)

Fig. 3A (uncropped)

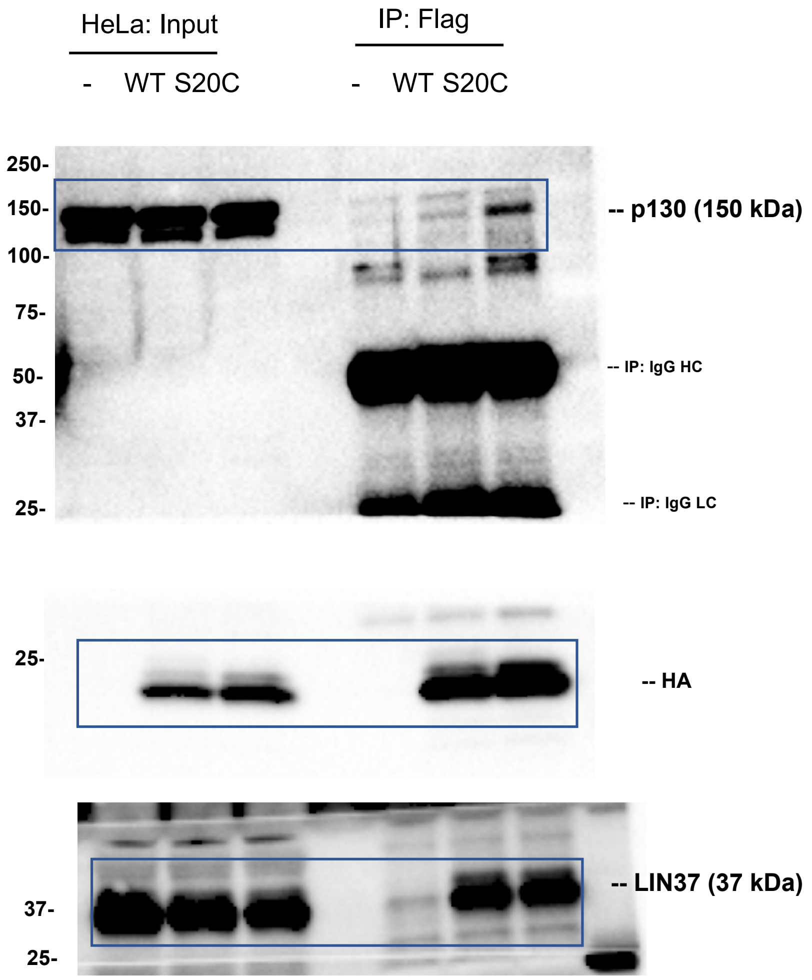

Fig. 3B (uncropped)

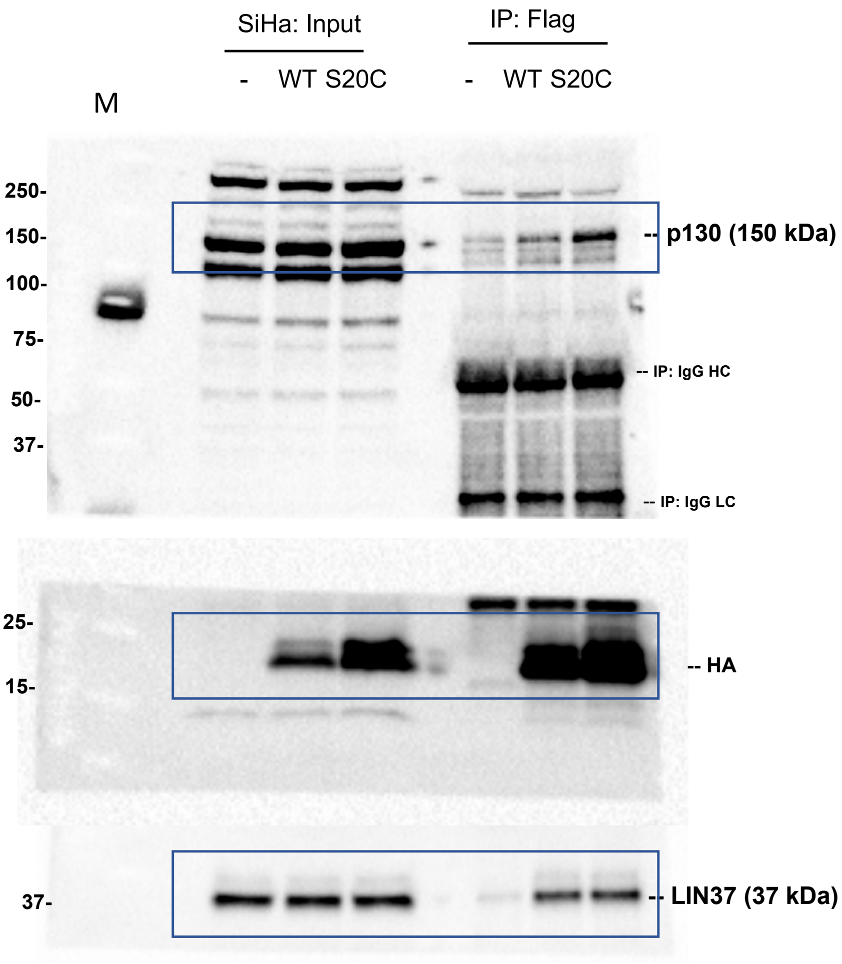

Fig. 3C, D (uncropped)

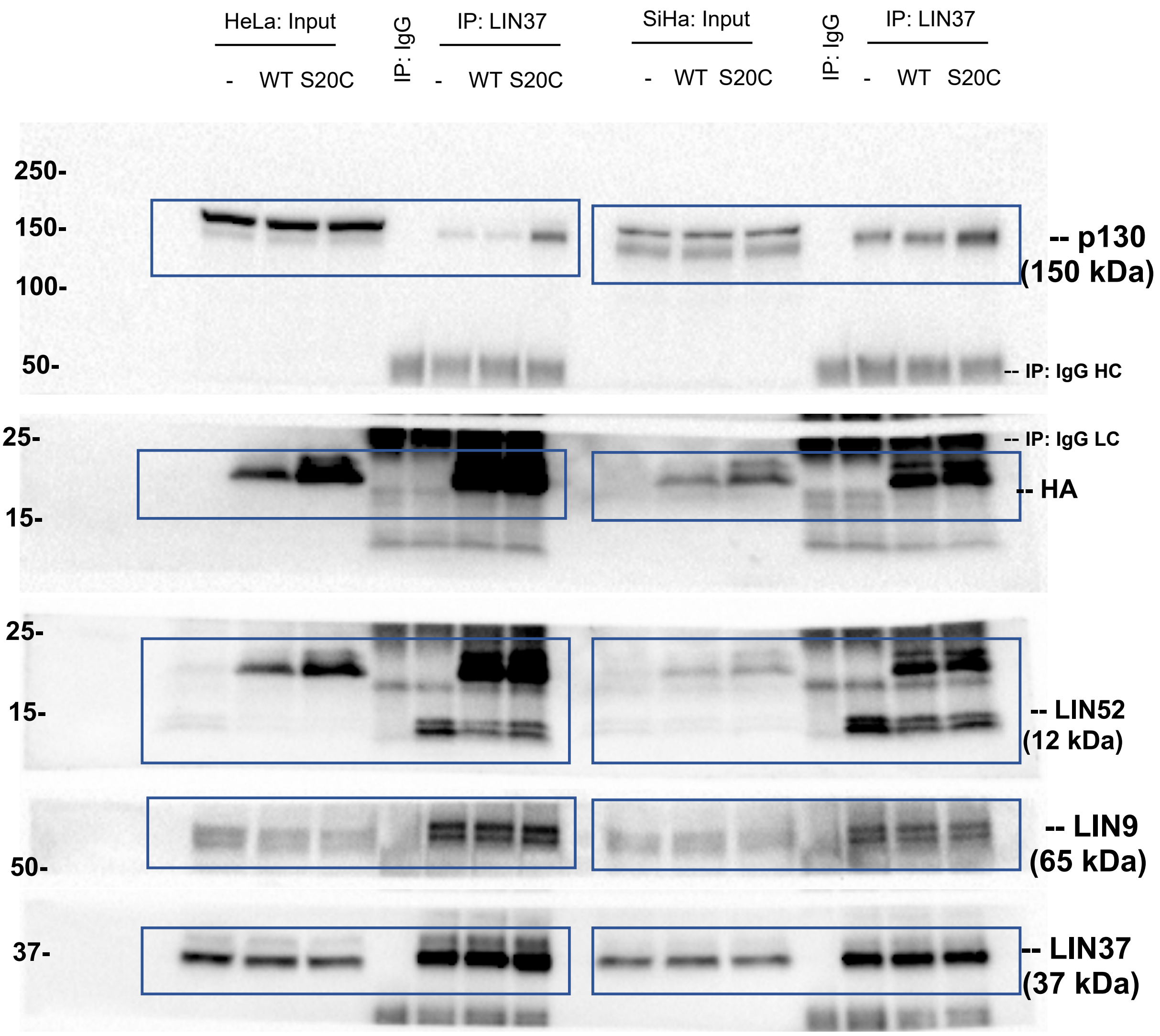

Fig. 3E (uncropped)

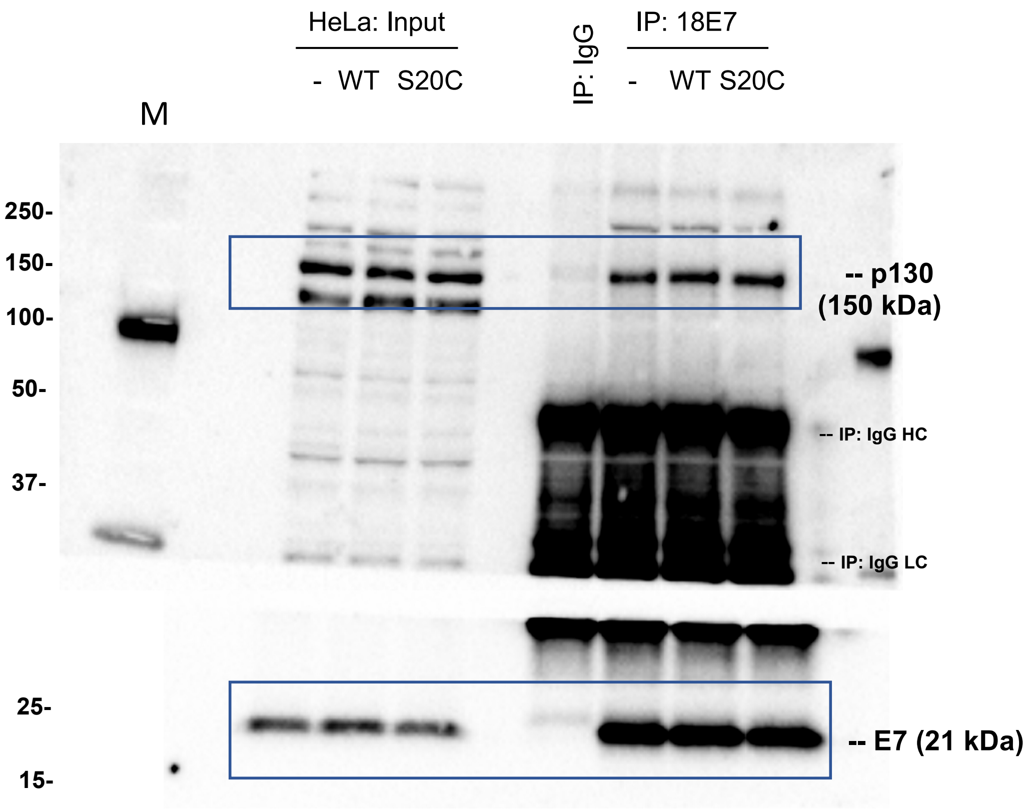

Fig. 3F (uncropped)

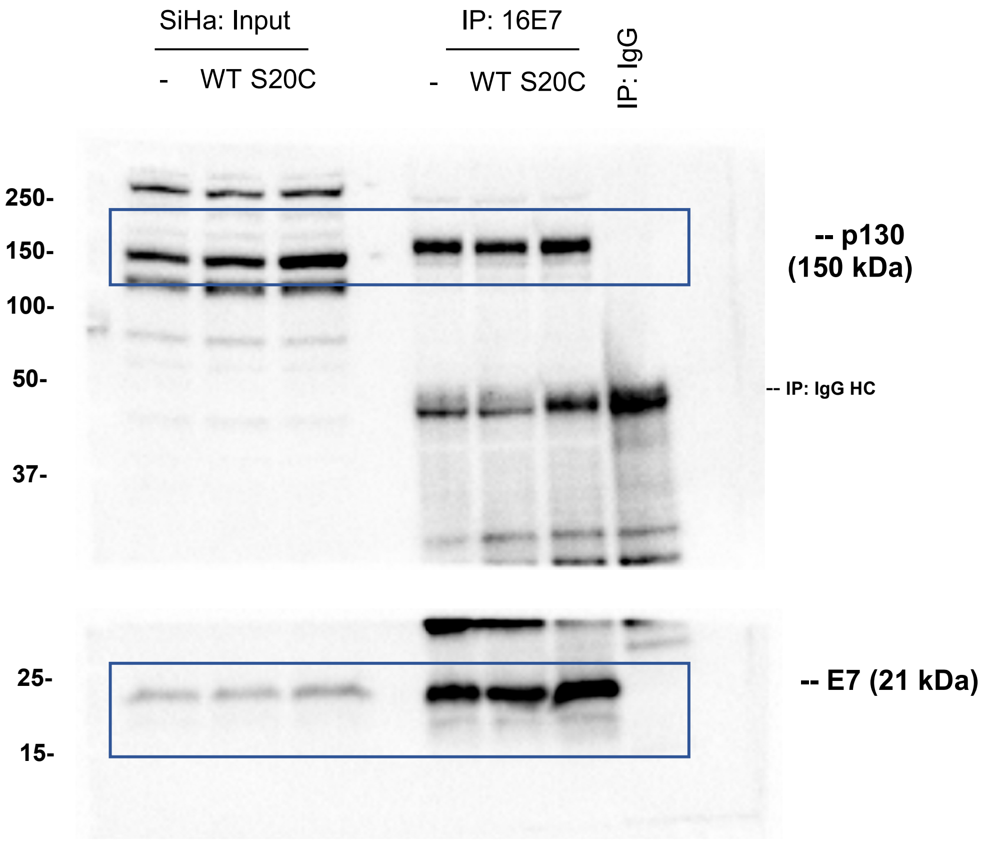

Supplement: Supplementary file 1 [file cancers-13-00489-s001.zip › cancers-1046677-supplementary.pdf]
